# Importance of lymph node immune responses in MSI-H/dMMR colorectal cancer

**DOI:** 10.1172/jci.insight.137365

**Published:** 2021-05-10

**Authors:** Koji Inamori, Yosuke Togashi, Shota Fukuoka, Kiwamu Akagi, Kouetsu Ogasawara, Takuma Irie, Daisuke Motooka, Yoichi Kobayashi, Daisuke Sugiyama, Motohiro Kojima, Norihiko Shiiya, Shota Nakamura, Shoichi Maruyama, Yutaka Suzuki, Masaaki Ito, Hiroyoshi Nishikawa

**Affiliations:** 1 Division of Cancer Immunology, Research Institute/Exploratory Oncology Research & Clinical Trial Center (EPOC), National Cancer Center, Tokyo/Chiba, Japan.; 2 Department of Colorectal Surgery, National Cancer Center Hospital East (NCCHE), Chiba, Japan.; 3 Surgery 1, Divisions of cardiovascular, Thoracic, General Endoscopic and Breast Surgery, Hamamatsu University School of Medicine, Hamamatsu, Japan.; 4 Division of Molecular Diagnosis and Cancer Prevention, Saitama Cancer Center (SCC), Saitama, Japan.; 5 Department of Immunobiology, Institute of Development, Aging and Cancer, Tohoku University, Sendai, Japan.; 6 Research Institute for Microbial Diseases, Osaka University, Osaka, Japan.; 7 Department of Immunology and; 8 Department of Nephrology, Nagoya University Graduate School of Medicine, Nagoya, Japan.; 9 Division of Pathology, NCCHE, Chiba, Japan.; 10 Department of Medical Genome Sciences, Graduate School of Frontier Sciences, The University of Tokyo, Chiba, Japan.

**Keywords:** Oncology, Cancer immunotherapy, T cell receptor, T cells

## Abstract

Patients with colorectal cancers (CRCs) generally exhibit improved survival through intensive lymph node (LN) dissection. However, recent progress in cancer immunotherapy revisits the potential importance of regional LNs, where T cells are primed to attack tumor cells. To elucidate the role of regional LN, we investigated the immunological status of nonmetastatic regional LN lymphocytes (LNLs) in comparison with those of the tumor microenvironment (tumor-infiltrating lymphocytes; TILs) using flow cytometry and next-generation sequencing. LNLs comprised an intermediate level of the effector T cell population between peripheral blood lymphocytes (PBLs) and TILs. Significant overlap of the T cell receptor (TCR) repertoire was observed in microsatellite instability–high/mismatch repair–deficient (MSI-H/dMMR) CRCs with high tumor mutation burden (TMB), although limited TCRs were shared between nonmetastatic LNs and primary tumors in microsatellite stable/MMR proficient (MSS/pMMR) CRC patients with low TMB. In line with the overlap of the TCR repertoire, an excessive LN dissection did not provide a positive impact on long-term prognosis in our MSI-H/dMMR CRC cohort (*n* = 130). We propose that regional LNs play an important role in antitumor immunity, particularly in MSI-H/dMMR CRCs with high TMB, requiring care to be taken regarding excessive nonmetastatic LN dissection in MSI-H/dMMR CRC patients.

## Graphical abstract

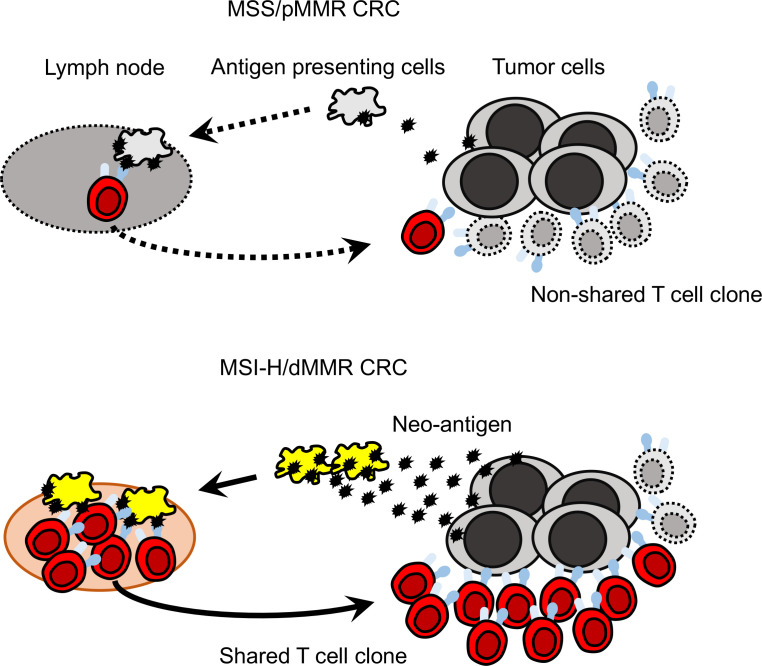

## Introduction

Colorectal cancers (CRCs) are the third most common cancer and the fourth most common cause of death from cancer in the world, accounting for approximately 1.1 million new cases and 550,000 deaths per year (1). Approximately 20% of CRC patients have distant metastases at the time of diagnosis. Despite recent progresses in treatment, including molecular-targeted therapy, patients suffering from metastatic CRCs have a poor 5-year survival rate of about 10% (2). More effective therapies, therefore, are urgently required.

Cancer immunotherapy, including immune checkpoint blockade (ICB), has been proven to be beneficial for patients with various types of cancer, including malignant melanoma and non–small cell lung cancer (3–5). Among gastrointestinal cancers, a recent phase III trial with an anti–PD-1 mAb for patients with advanced gastric cancers (GC) or esophageal cancers (EC) showed a survival benefit, resulting in the approval of anti–PD-1 mAb for treating GC or EC in Japan (6, 7). However, the efficacy of anti–PD-1 mAb is not satisfactory, and ICB seems to be less effective against metastatic CRCs, especially microsatellite stable/mismatch repair proficient (MSS/pMMR) CRCs (8, 9). By contrast, PD-1 blockade was reported to be effective against microsatellite instability–high/MMR-deficient (MSI-H/dMMR) CRCs, with a response rate of 30%–70%, which can be explained by the high tumor mutation burden (TMB), potentially becoming tumor neoantigens (8, 10, 11), although MSI-H/dMMR accounts for approximately 10% of CRCs (12). Therefore, studies to understand the detailed immunological features of CRCs, particularly in the tumor microenvironment (TME), are needed to develop better treatment strategies.

The existence of lymph node (LN) metastases is important in deciding the treatment strategy for CRCs, and patients who undergo an intensive LN dissection exhibit improved survival (13, 14). One of the reasons for LN dissection is to accurately determine the disease stage. Accurate staging is a critical issue to consider whether further treatments are required. According to some guidelines, examination of a minimum of 12 LNs is recommended for the accurate staging (13, 14). On the other hand, from an immunological view, regional LNs are considered to play an essential role in antitumor immunity because T cells are generally primed by antigen presenting cells (APCs) that capture tumor antigens in tumor tissues and infiltrate into regional LN (15, 16). Therefore, an excessive LN dissection, especially of nonmetastatic LNs, may lead to negative effects regarding antitumor immune responses. To reconcile this dilemma of cancer treatment, a broad T cell landscape in the TME, LNs, and peripheral blood should be elucidated in humans, since it is difficult to recapitulate the landscape of immune responses with animal models, particularly with transplanted tumors. Here, we comprehensively investigated the immunological status of nonmetastatic regional LNs (LN lymphocytes; LNLs) in comparison with those of the TME (tumor-infiltrating lymphocytes; TILs) and peripheral blood (peripheral blood lymphocytes; PBLs) in CRC patients to further understand the immunological features that lead to optimal CRC therapy.

## Results

### LNLs exhibit an intermediate phenotype between PBLs and TILs, especially for effector memory T cells.

**Figure 1:**
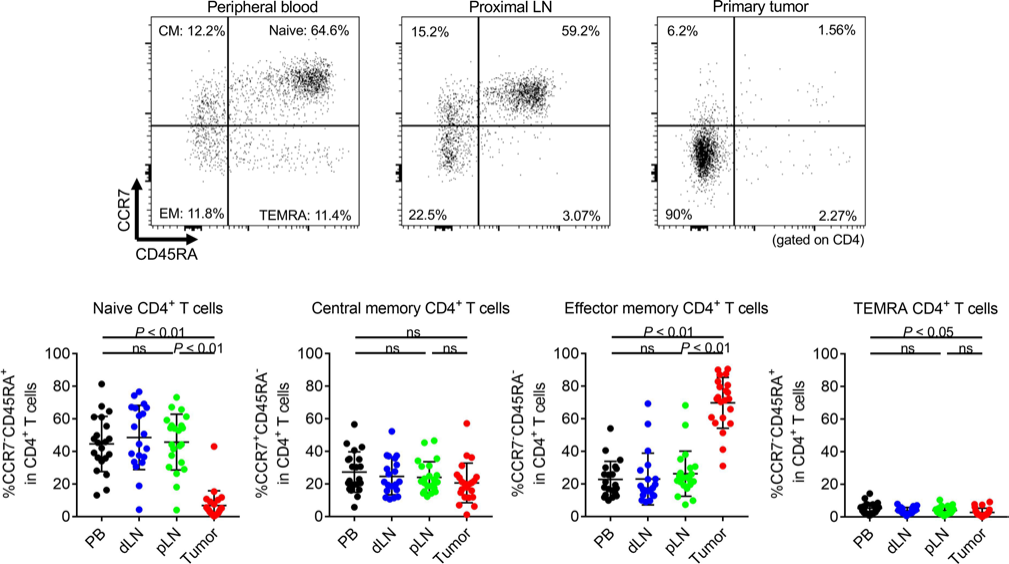
Differences of CD4^+^ T cell subpopulations among peripheral blood, LNs, and primary tumors. PBLs, LNLs, and TILs from 21 CRC patients who received surgical resection were prepared, and immunological phenotypes were examined with flow cytometry. Representative flow cytometry staining (upper) and summaries (lower) for the frequency of CCR7^+^CD45RA^+^CD4^+^ T cells (naive), CCR7^+^CD45RA^–^CD4^+^ T cells (central memory), CCR7^–^CD45RA^–^CD4^+^ T cells (effector memory), and CCR7^–^CD45RA^+^CD4^+^ T cells (terminally differentiated effector memory) in conventional CD4^+^ T cells of PBLs, LNLs, and TILs. Means ± SDs are shown, and statistical analyses were performed using 1-way ANOVA with Bonferroni corrections. CM, central memory; EM, effector memory; TEMRA, terminally differentiated effector memory; PB, peripheral blood; dLN, distal LN; pLN, proximal LN.

**Figure 2:**
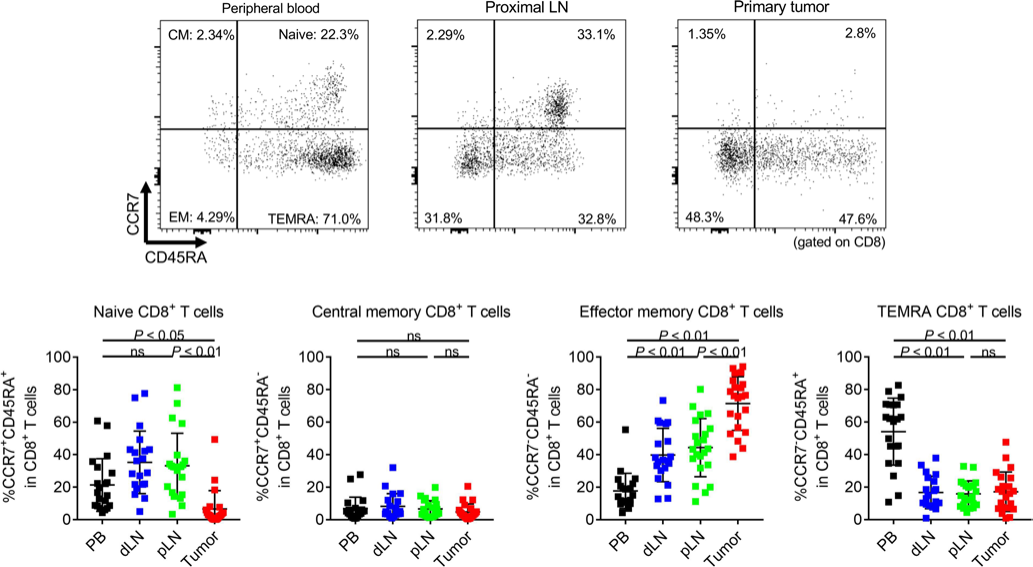
Differences of CD8^+^ T cell subpopulations among peripheral blood, LNs, and primary tumors. PBLs, LNLs, and TILs from 21 CRC patients who received surgical resection were examined with flow cytometry as in Figure 1. Representative flow cytometry staining (upper) and summaries (lower) for the frequency of CCR7^+^CD45RA^+^CD8^+^ T cells (naive), CCR7^+^CD45RA^–^CD8^+^ T cells (central memory), CCR7^–^CD45RA^–^CD8^+^ T cells (effector memory), and CCR7^–^CD45RA^+^CD8^+^ T cells (terminally differentiated effector memory) in conventional CD8^+^ T cells of PBLs, LNLs, and TILs. Means ± SDs are shown, and statistical analyses were performed using 1-way ANOVA with Bonferroni corrections. CM, central memory; EM, effector memory; TEMRA, terminally differentiated effector memory; PB, peripheral blood; dLN, distal LN; pLN, proximal LN.

We first addressed immunological phenotypes in CRC patients from whom sufficient amounts of PBLs, LNLs, and TILs were available (NCCHE cohort). The clinical characteristics of 21 CRC patients enrolled in this study are summarized in Supplemental Table 1 (supplemental material available online with this article; https://doi.org/10.1172/jci.insight.137365DS1). One patient had double cancer of the transverse colon (C416_T) and sigmoid colon (C416_S), both of which were subjected to assessment. There were 5 right-sided tumors and 6 left-sided tumors in pMMR CRCs, and there were 8 right- and 3 left-sided tumors in dMMR CRCs. All patients received LN dissection, regardless of tumor location, according to the Japanese Society for Cancer of the Colon and Rectum (JSCCR) guidelines (17), and the number of LN dissection was not significantly different with location (Supplemental Figure 1A). Each dissected LN was halved with the maximum surface, all of which were pathologically examined. If there were pathological metastases including micrometastases in LNs, such LNs were excluded from the analyses to avoid including metastatic LNs in our assay. CD8^+^ T cell infiltration was higher in early-stage or dMMR CRCs compared with late-stage or pMMR CRCs, as expected (Supplemental Figure 2). The frequency of effector memory CD8^+^ T cells in LNLs tended to be higher, though not significantly higher, in dMMR CRCs (Supplemental Figure 3). TCGA analyses have shown that a lot of genes related to cytotoxic activity (*GZMA* and *PRF1*) and T cell exhaustion (*PDCD1*, *LAG3*, *HAVCR2*, etc.) exhibited significantly high expression in MSI-H/dMMR CRCs and that MSI-H/dMMR CRCs contained abundant CD8^+^ T cell infiltration from CIBERSORTx (Supplemental Figure 4) (12, 18). In comparison with PBLs, LNLs, and TILs, CD4^+^ T cell proportion was the highest in LNLs, followed by PBLs and TILs. By contrast, the CD8^+^ T cell proportion tended to be higher in TILs, followed by PBLs and LNLs (Supplemental Figure 5). T cells generally infiltrate into local tissues after priming at draining LNs, where APCs present cognate antigens (16). In accordance with this, TILs dominantly contained CCR7^–^CD45RA^–^ cells (effector memory T cells), whereas limited CCR7^+^CD45RA^+^ T cells (naive T cells) were observed compared with PBLs and LNLs (Figure 1 and Figure 2). LNLs comprised a similar level of the naive T cell population as PBLs and an intermediate level between PBLs and TILs for effector memory T cells in both CD4^+^ and CD8^+^ T cells. Distal LNLs were comparable with proximal LNLs in both CD4^+^ and CD8^+^ T cells (Figure 1 and Figure 2). Surgically resected tonsils by chronic tonsillitis were provided as another cohort of control, showing significant differences in many populations from LNs that we analyzed in our CRC cohort (Supplemental Figure 6).

### Activation status of LNLs is in an intermediate range between PBLs and TILs.

**Figure 3:**
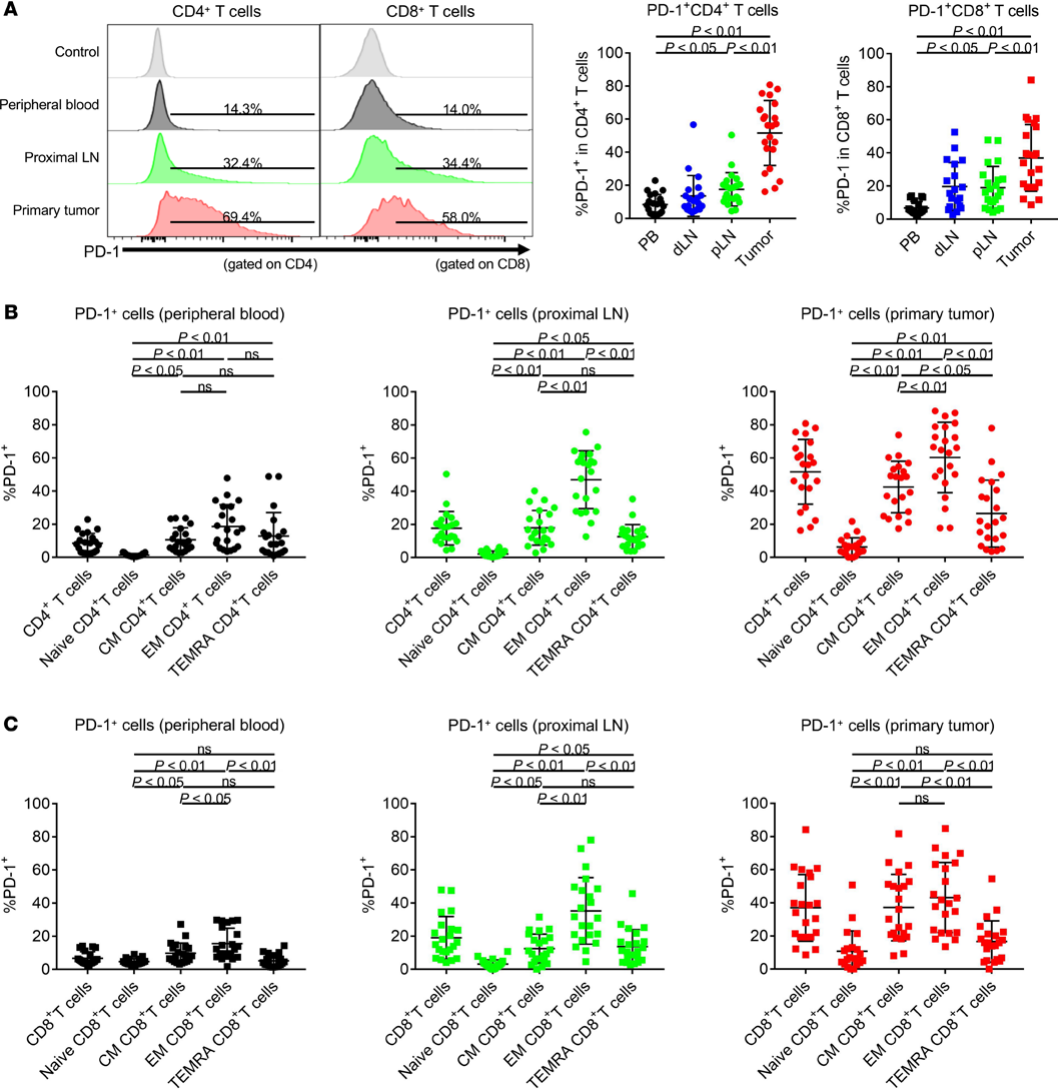
PD-1 expression by T cells in PBLs, LNLs, and TILs. PBLs, LNLs, and TILs from 21 CRC patients who received surgical resection were examined with flow cytometry as in Figure 1. (**A**) Representative flow cytometry staining (left) and summaries (right) for the frequency of PD-1–expressing cells in CD4^+^ and CD8^+^ T cells in PBLs, LNLs, and TILs. (**B**) Summaries for the frequency of PD-1–expressing cells in total CD4^+^ T cells, CCR7^+^CD45RA^+^CD4^+^ T cells (naive), CCR7^+^CD45RA^–^CD4^+^ T cells (central memory), CCR7^–^CD45RA^–^CD4^+^ T cells (effector memory), and CCR7^–^CD45RA^+^CD4^+^ T cells (terminally differentiated effector memory) in PBLs, LNLs, and TILs. (**C**) Summaries for the frequency of PD-1–expressing cells in total CD8^+^ T cells, CCR7^+^CD45RA^+^CD8^+^ T cells (naive), CCR7^+^CD45RA^–^CD8^+^ T cells (central memory), CCR7^–^CD45RA^–^CD8^+^ T cells (effector memory), and CCR7^–^CD45RA^+^CD8^+^ T cells (terminally differentiated effector memory) in PBLs, LNLs, and TILs. Means ± SDs are shown, and statistical analyses were performed using 1-way ANOVA with Bonferroni corrections. CM, central memory; EM, effector memory; TEMRA, terminally differentiated effector memory; PB, peripheral blood; dLN, distal LN; pLN, proximal LN.

We further examined the T cell activation status according to PD-1 expression, since PD-1 expression is induced upon T cell receptor (TCR) stimulation and is associated with clinical responses by PD-1 blockade in some cancer types (19–21). PD-1 was highly expressed by TILs, followed by LNLs and PBLs, in both CD4^+^ and CD8^+^ T cells (Figure 3A). PD-1 expression was the highest in CCR7^–^CD45RA^–^ effector memory T cells in PBLs, LNLs, and TILs (Figure 3, B and C). These findings suggest that PD-1 expression may reflect the activation status of tumor antigen-specific T cells.

**Figure 4:**
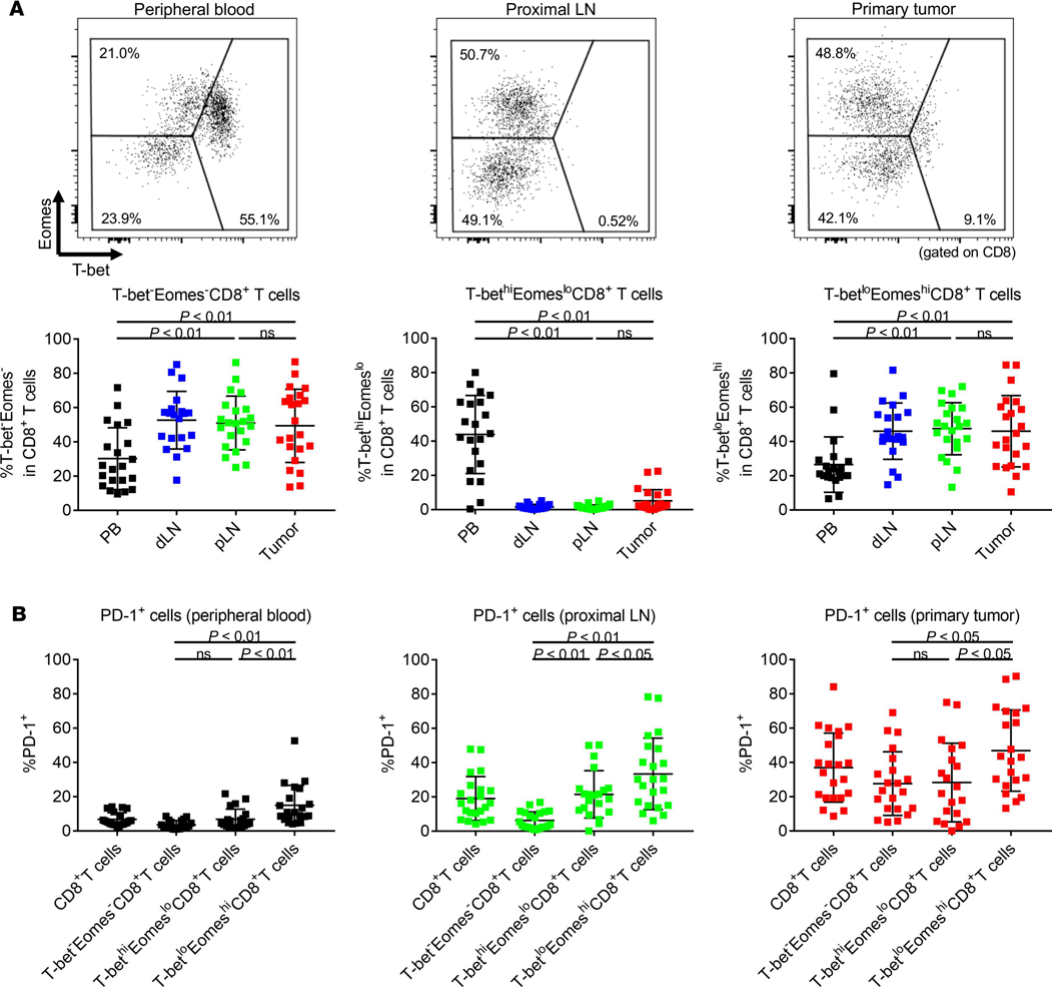
The expression of transcription factors (T-bet and Eomes) in PBLs, LNLs, and TILs. PBLs, LNLs, and TILs from 21 CRC patients who received surgical resection were examined with flow cytometry as in Figure 1. (**A**) Representative flow cytometry staining (upper) and summaries (lower) for the frequency of T-bet– and Eomes–expressing cells in CD8^+^ T cells in PBLs, LNLs, and TILs. T cells were divided into 3 fractions based on the expression of T-bet and Eomes: T-bet^–^Eomes^–^, T-bet^hi^Eomes^lo^, and T-bet^lo^Eomes^hi^. (**B**) PD-1 expression according to T-bet and Eomes expression in CD8^+^ T cells in PBLs, LNLs, and TILs. Means ± SDs are shown, and statistical analyses were performed using 1-way ANOVA with Bonferroni corrections. PB, peripheral blood; dLN, distal LN; pLN, proximal LN.

To gain further insight into the high PD-1 expression in TILs, we explored the expression of T-bet and Eomes, since these transcription factors are reportedly associated with PD-1 expression (22, 23). We divided T cells into 3 fractions: T-bet^–^Eomes^–^, T-bet^hi^Eomes^lo^, and T-bet^lo^Eomes^hi^ (Figure 4A). There were significant differences in each population among PBLs, LNLs, and TILs in CD8^+^ T cells. T-bet^lo^Eomes^hi^CD8^+^ T cells were significantly higher in LNLs and TILs than those in PBLs, although more than half of PBLs were composed of T-bet^hi^Eomes^lo^CD8^+^ T cells (Figure 4A). PD-1 expression was the highest in T-bet^lo^Eomes^hi^CD8^+^ T cells, followed by T-bet^hi^Eomes^lo^CD8^+^ T cells and T-bet^–^Eomes^–^CD8^+^ T cells in PBLs, LNLs, and TILs, which was consistent with previous reports (Figure 4B) (22, 23). Interestingly, PD-1 was noticeably expressed by TILs even in T-bet^–^Eomes^–^CD8^+^ T cells, suggesting that other factors may be related to the high PD-1 expression in TILs in addition to T-bet and Eomes. By contrast, the T-bet^–^Eomes^–^ population was comparably dominant in CD4^+^ T cells from PBLs, LNLs, and TILs (Supplemental Figure 7). The activation statuses of TILs, LNLs, and PBLs in humans reflect the hypothesis developed by animal models; T cells that are activated in draining LNs infiltrate into tumor tissues.

### Immune suppressive FOXP3^+^CD4^+^ T cells are abundant in LNLs.

**Figure 5:**
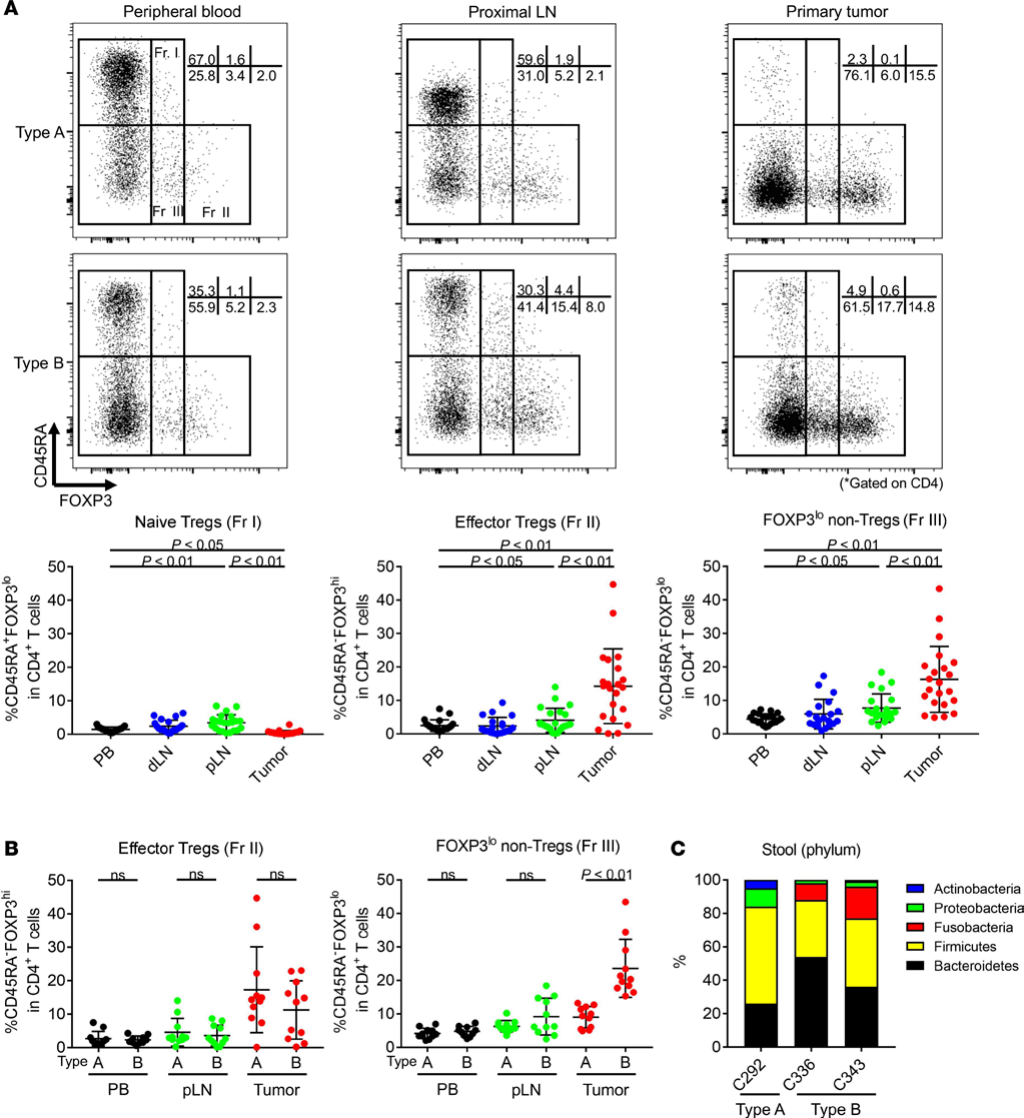
Differences of FOXP3^+^CD4^+^ T cell subpopulations in PBLs, LNLs, and TILs. PBLs, LNLs, and TILs from 21 CRC patients who received surgical resection were examined with flow cytometry as in Figure 1. (**A**) Representative flow cytometry staining (upper) and summaries (lower) for the frequency of FOXP3^+^CD4^+^ T cell populations in PBLs, LNLs, and TILs. CRCs are classified into 2 types according to FOXP3^lo^ non-Treg infiltration in the TME: type A (low FOXP3^lo^non-Treg) and B (high FOXP3^lo^non-Treg). (**B**) Differences in the frequency of eTregs and FOXP3^lo^ non-Tregs according to CRC subtypes. (**C**) Fecal metagenome was analyzed by 16S rRNA sequencing. *Fusobacteria* was frequently found in stools from type B. Means ± SDs are shown, and statistical analyses were performed using 1-way ANOVA with Bonferroni corrections in **A** and the *t* tests in **B**. PB, peripheral blood; dLN, distal LN; pLN, proximal LN.

We next interrogated immune suppressive cells, particularly CD4^+^ Tregs, since Tregs inhibit antitumor immunity and contribute to unfavorable clinical courses in CRCs (24). Correctly identifying CD4^+^ Tregs in humans is compromised due to the upregulation of FOXP3, the master transcription factor of Tregs, upon TCR stimulation in conventional T cells (25). We therefore proposed a classification of human Tregs based on the expression levels of a naive marker, CD45RA and FOXP3. FOXP3^+^CD4^+^ T cells were able to be divided into 3 fractions (Fr): naive Tregs (Fr I: CD45RA^+^FOXP3^lo^CD4^+^); effector Tregs (eTregs) (Fr II: CD45RA^–^FOXP3^hi^CD4^+^) with a strong immune suppressive function; and cytokine-producing non-Tregs (Fr III: CD45RA^–^FOXP3^lo^CD4^+^) without a suppressive function (Figure 5A) (24, 26, 27). eTregs have higher expression of CTLA-4 — which is an important immune checkpoint molecule and plays a crucial role in Treg-mediated immune suppression (28) — than that of FOXP3^lo^ non-Tregs (Supplemental Figure 8). In addition, other Treg-related molecules, such as CD39, ICOS, and GITR (27), were highly expressed by eTregs (Supplemental Figure 8). These data support the notion that eTregs are bona fide Tregs with an immune suppressive function and that FOXP3^lo^ non-Tregs are different from immune suppressive Tregs (24, 26, 27).

The frequencies of eTregs and FOXP3^lo^ non-Tregs were significantly higher in TILs than those in PBLs, while naive Tregs were more abundant in PBLs than in TILs (Figure 5A). Similarly, the frequency of CD45RA^–^FOXP3^–^CD4^+^ T cells was significantly higher in TILs than in PBLs, whereas the frequency of CD45RA^+^FOXP3^–^CD4^+^ T cells was significantly higher in PBLs than in TILs (Supplemental Figure 9). The frequencies of eTregs and FOXP3^lo^ non-Tregs in LNLs were in the intermediate range between PBLs and TILs, whereas naive Tregs in LNLs were highly detected compared with those in PBLs and TILs (Figure 5A). eTregs were frequently found in TILs and LNLs of CRCs located in left side of the colon (Supplemental Figures 2 and 3). We have previously proposed a potentially novel classification of CRCs according to FOXP3^lo^ non-Treg infiltration in the TME: type A (low FOXP3^lo^non-Treg) and B (high FOXP3^lo^non-Treg) (24). CRCs with abundant infiltration of non-Tregs (type B) showed a significantly better prognosis than those with predominant eTreg infiltration. Tumor invasion by intestinal bacteria, especially *Fusobacterium nucleatum*, induced the development of such inflammatory non-Tregs (24). Interestingly, the frequency of FOXP3^lo^ non-Tregs in LNLs was variable in type A (*n* = 11) and type B (*n* = 11), although there was a trend to be higher, particularly in 4 patients, in type B than in type A (Figure 5, A and B). Furthermore, *Fusobacteria* was found in stools from 2 type B CRC patients with high FOXP3^lo^ non-Tregs in LNLs (Figure 5C). These findings suggest that the frequency of eTregs in LNLs is detected at an intermediate level between that of PBLs and TILs and that FOXP3^lo^ non-Tregs may be induced by inflammation caused by *Fusobacteria* — not only in TILs, but also in LNLs.

### TCR repertoire is shared between TILs and LNLs in dMMR CRC patients.

**Figure 6:**
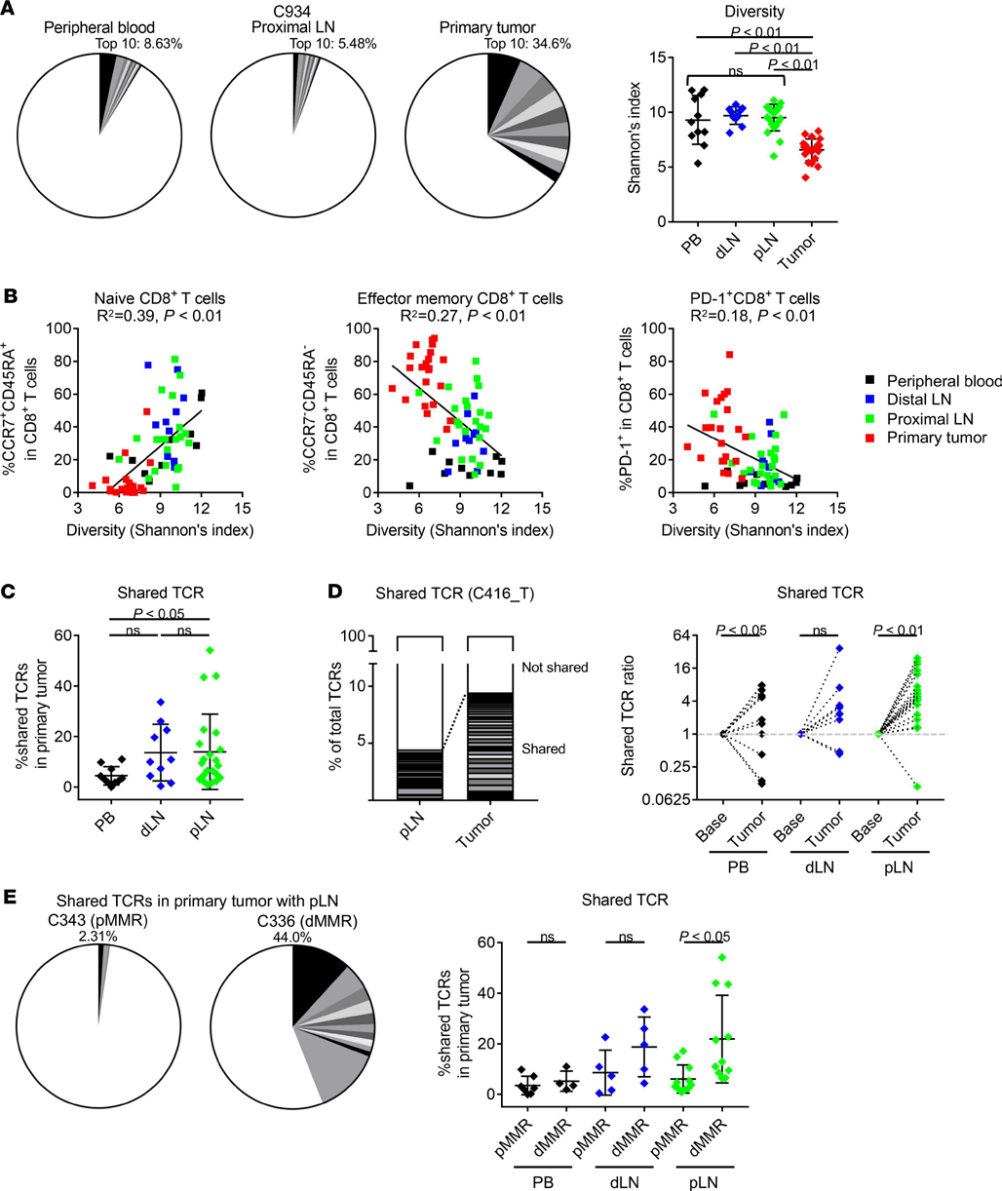
TCR repertoire among PBLs, LNLs, and TILs. PBLs, LNLs, and TILs from 21 CRC patients who received surgical resection were prepared, and the TCR sequencing was performed with next-generation sequencing. (**A**) Diversity of the TCR repertoire in PBLs, LNLs, and TILs evaluated with Shannon’s index. The top 10 TCRs are colored (left), and the summary of Shannon’s index is shown (right). (**B**) Correlation between TCR diversity and CCR7^+^CD45RA^+^CD8^+^ T cell (naive), CCR7^–^CD45RA^–^CD8^+^ T cell (effector memory), or PD-1^+^CD8^+^ T cell proportion. (**C**) Shared TCRs in TILs with PBLs or LNLs. The frequency of shared TCRs in TILs with PBLs or LNLs is shown. (**D**) TCR expansion from PBLs or LNLs to TILs. The frequency of shared TCRs in LNLs and TILs (left, representative patient) and the summary of shared TCR ratio (right) are shown. Shared TCRs are colored. (**E**) The frequency of shared TCRs in TILs with PBLs or LNLs according to MMR status. Pie charts of a representative pMMR CRC patient and a representative dMMR CRC patient (left) and the summary of shared TCR (right) are presented. Shared TCRs are colored. Means ± SDs are shown, and statistical analyses were performed using 1-way ANOVA with Bonferroni corrections in **A** and **C**, and *t* tests in **D** and **E**. PB, peripheral blood; dLN, distal LN; pLN, proximal LN.

As the frequencies and immunological phenotypes of effector T cells and immune suppressive cells were in an intermediate range in LNLs, we asked whether T cells activated in draining LNs infiltrated into tumor tissues via exploring shared TCR-β between LNs and primary tumors. TCR diversity, which was evaluated with Shannon’s index, was significantly higher in PBLs and LNLs than that in TILs (Figure 6A). While TCR repertoires of naive CD8^+^ T cells harbored high TCR diversity, TCR repertoires of activated CD8^+^ T cells (detected as effector memory CD8^+^ T cells and PD-1^+^CD8^+^ T cells) in tumors was significantly skewed (Figure 6B). Together with previous reports (19, 21), we therefore propose that the skewing of T cell clones, particularly PD-1^+^CD8^+^ T cells in the TME, reflect the activation of tumor antigen-specific T cells. Some TCRs were shared between PBLs or LNLs and TILs, and shared TCRs with LNLs were frequently found in TILs compared with those with PBLs (Figure 6C). These shared TCRs were expanded from PBLs or LNLs to TILs in many patients, especially from proximal LNs to primary tumors (Figure 6D). Ten CRC samples harbored a considerable level of shared TCRs in primary tumors with proximal LNs (>10%), 7 of which were observed in patients with dMMR CRCs. Remaining dMMR CRCs had intermediately shared TCRs with proximal LNs (5%–10%) (Figure 6E). By sharp contrast, more than half of pMMR CRC samples had few shared TCRs with proximal LNs (<5%) (Figure 6E). There was no significant difference in TCR diversity of PBLs, LNLs, or TILs between pMMR and dMMR CRCs, despite a significant difference in shared TCRs (Supplemental Figure 10A). Although we analyzed nonmetastatic LNs, these data were also examined according to pathological staging (pStage) because LN metastases might affect nonmetastatic LNs. There was no significant difference in TCR diversity or shared TCRs between pStage I or II (pN0) and pStage III or IV (pN1–2) (Supplemental Figure 10B). The frequency of the shared TCRs in TILs with distal LNLs showed a trend to be lower than that with proximal LNLs, although the difference was not significant due to the small number (Figure 6C). These findings suggest that a variety of T cell clones activated in regional, especially proximal, LNs infiltrate into the TME of MSI-H/dMMR CRC and attack tumor cells.

Additionally, we performed whole exon sequencing (WES) for CRC samples with sufficient volume and found that dMMR CRC samples had significantly higher TMB, including both nonsynonymous single nucleotide variations and insertion/deletion (Supplemental Figure 11A). There was no significant association between TCR diversity and TMB either in proximal LN or in primary tumor (Supplemental Figure 11B). Abundant shared TCRs between proximal LNs and primary tumors, although not significant, were detected in higher TMB samples (Supplemental Figure 11C). The TCR repertoire was highly overlapped between TILs and LNLs in dMMR CRC patients with high TMB, but not in patients with pMMR CRCs with low TMB, which can be explained by the presence of antigen-specific T cells against neo-antigens derived from gene alterations.

### Intensive LN dissection may induce negative impacts on prognosis in patients with MSI-H/dMMR CRCs.

**Figure 7:**
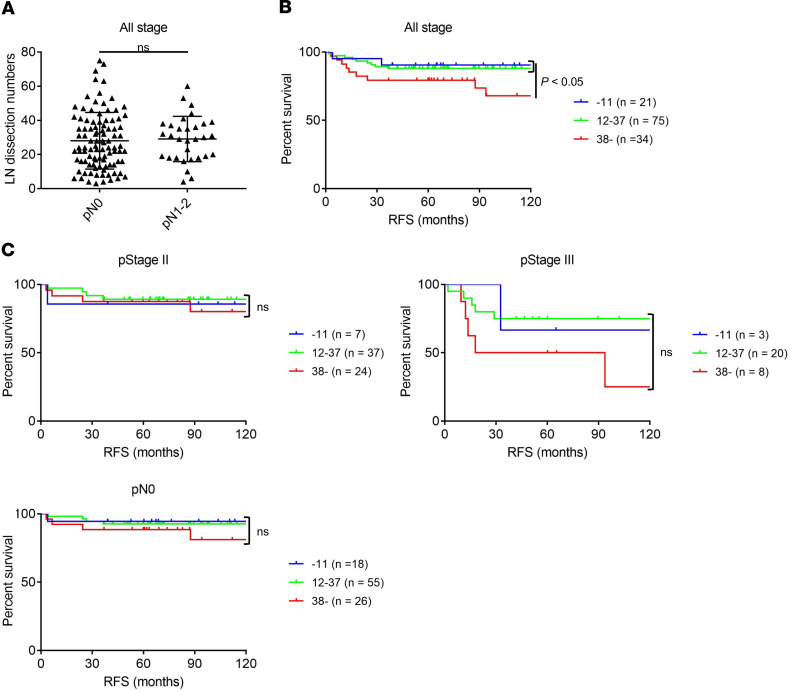
Correlation between LN dissection number and recurrence after surgery in our MSI-H cohort (SCC cohort). (**A**) LN dissection number based on pathological CRC staging. Means ± SDs are shown, and statistical analyses were performed using *t* test. (**B**) RFS in the MSI-H cohort based on the number of LNs dissected in all pStage. (**C**) RFS in the MSI-H cohort based on the number of LNs dissected in each pStage. The RFS was investigated with the Kaplan-Meier method and was compared among groups using the log-rank test.

Many patients with dMMR CRCs harbored substantially shared TCRs between LNLs and TILs, while most patients with pMMR CRCs showed a limited overlap. Thus, we hypothesized that regional LNs could play an important role in developing antitumor immunity, particularly in patients with MSI-H/dMMR CRCs compared with those with MSS/pMMR CRCs. Then, the prognostic significance of the number of dissected LNs was investigated in another MSI-H cohort (SCC cohort) (Supplemental Table 2). The characteristics of 130 patients with MSI-H/dMMR CRCs, such as female, right side, early stage, and poor differentiation, were similar to previous reports (29–31). No significant difference in LN dissection number was observed according to tumor location (right versus left), as well as NCCHE CRC cohort, from which we collected TILs and LNLs (Supplemental Figure 1B). Because some guidelines have recommended examination of a minimum of 12 LNs (13, 14), only 21 CRC patients in the cohort received LN dissection with less than 12, and the number of LNs dissected in patients with pN1–2 was comparable with that in patients with pN0, supporting the accurate staging in our cohort (Figure 7A). Indeed, the presence of LN metastases in patients with LN dissection number < 12 was comparable with that in patients with ≥ 12 (3 of 21 versus 28 of 109, *P* = 0.41). This can also be demonstrated by the reduced incidence of LN metastases in MSI-H/dMMR CRCs, which is different from MSS/pMMR CRCs (32, 33). A receiver operating characteristic (ROC) curve determined the cut-off value of the number of excessive LN dissection as 38 (Supplemental Figure 12). The presence of LN metastases in patients with LN dissection number < 38 was also comparable with that in patients with ≥ 38 (23 of 96 versus 8 of 34, *P* > 0.99). In all stages, the small number of LN dissection (<12) was not related to the unfavorable prognosis (Figure 7B). Interestingly, the high number of LN dissection (≥38) exhibited a significantly shorter recurrence-free survival (RFS) compared with the low number of LN dissection (<38) (Figure 7B). We next focused on patients with each pStage: no patient experienced recurrence in pStage 0 or I. In pStage II, the number of dissected LNs was not correlated with RFS, although the number of LN dissection is reportedly a prognostic factor in pMMR CRCs (Figure 7C) (13, 14). Furthermore, in pStage III and pN0, the high number of dissected LNs (≥38) corresponded with a slightly shorter RFS, but the difference was not significant, compared with that of other groups (Figure 7C). Taken together, excessive LN dissection could provide a negative impact on long-term prognosis in patients with MSI-H/dMMR CRCs, by removing T cells that are activated in draining LNs and infiltrate into the TME to attack tumors.

## Discussion

While the importance of comprehensive analyses from tumor tissues to the periphery is appreciated based on the data from animal models, systemic evaluation has been limited in humans. Here, we extensively explored the immunological status of tumors, nonmetastatic LNs, and peripheral blood and found a considerable overlap of TCRs between LNs and primary tumors in MSI-H/dMMR CRCs with high TMB. By contrast, very few shared TCRs between nonmetastatic LNs and primary tumors in MSS/pMMR CRC patients with low TMB was observed. Furthermore, in the MSI-H/dMMR cohort, excessive LN dissection could provide a negative impact on long-term prognosis, suggesting a need to be careful with intensive nonmetastatic LN dissection, particularly in MSI-H/dMMR CRC patients.

Approximately 10%–20% of CRCs are diagnosed as MSI-H/dMMR, with some ethnic variances, such as approximately 5% of Japanese CRC patients (34). The clinical characteristics of MSI-H/dMMR CRCs are related to younger age, right side colon, earlier stage (most commonly in stage II), female, and poor differentiation (29–31). Our MSI-H/dMMR cohort followed similar trends, such as early stage, female, and poor differentiation. Additionally, MSI-H/dMMR CRCs exhibited a favorable prognosis compared with that of MSS/pMMR tumors in early stage (32, 33), while MSI-H/dMMR advanced CRCs, including recurrence, seem to show a worse prognosis than MSS/pMMR advanced CRCs (35–37). These controversial findings could be explained from an immunological view (38); in early-stage MSI-H/dMMR CRCs, tumor-specific neo-antigens derived from gene alterations in MSI-H/dMMR CRCs elicit strong antitumor immune responses that correlate with a favorable prognosis (39, 40). Then, severe immune suppression and immune selection to escape antitumor immune responses are required for MSI-H/dMMR CRC progression, resulting in increases of the malignant potential associated with poor prognosis (41).

Accurate staging is critical for considering the proper treatment method. “More is better” has been well established, including in Japanese clinical settings for the accurate staging of CRCs. Accordingly, some guidelines have recommended examination of a minimum of 12 LNs (13, 14). T cells are generally primed at LNs and infiltrate into tumor tissues (15), although T cells could reportedly be primed and activated in tumor tissues (42). Thus, regional LNs often play an important role in developing antitumor immunity. Accordingly, if regional LNs are removed or lymphocyte migration from LNs is prevented, antitumor immunity fails to control tumor progression in mouse models (43, 44). In fact, considerably shared TCRs have been observed between LNs and primary tumors from MSI-H/dMMR CRCs with high TMB, potentially becoming tumor neo-antigens. One can therefore envision that intensive LN dissection may have negative effects on antitumor immunity — especially in MSI-H/dMMR CRCs with high TMB. Indeed, patients who received a limited LN dissection (the number of LNs dissected < 12) was not correlated with a shorter RFS. Rather, those who received an intensive LN dissection (the number of LNs dissected ≥ 38), who were expected to have more accurate staging compared with the others, exhibited a slightly shorter RFS, regardless of pStage, while limited LN dissection is reportedly associated with a poor prognosis in MSS/pMMR CRCs (13, 14). Thus, we need to be careful with excessive nonmetastatic LN dissections in MSI-H/dMMR CRCs with high TMB based on the following reasons: (a) the reduced incidence of LN metastases (32, 33), (b) substantial shared T cell clones between LNs and primary tumors, and (c) no influence of LN dissection number on the outcome after surgery and poor prognosis after recurrence (37) in MSI-H/dMMR CRCs. Furthermore, ICBs, which are reportedly effective against MSI-H/dMMR CRCs (8, 10, 11), could exhibit more efficacy in settings where regional LNs are remained, even after surgery or neo-adjuvant settings, because ICBs promote a new infiltration of tumor antigen-specific T cells (45) and because regional LNs may harbor such tumor antigen-specific T cells as observed in our study. Although the detailed role of PD-1^+^ T cells in LNs remains unclear, recent mouse studies have demonstrated that LNs contained enriched PD-1^+^ tumor–specific progenitor T cells and that those PD-1^+^ T cells play an important role in PD-1 blockade–mediated antitumor immunity (46, 47). By contrast to MSI-H/dMMR CRCs, patients with CRCs, most of which consist of MSS/pMMR, reportedly show a better prognosis after intensive LN dissection, meaning that a larger LN dissection leads to accurate staging and reduced residual lesions (13, 14). This is consistent with our present study showing that very few shared TCRs between nonmetastatic LNs and primary tumors were observed.

It is important to correctly identify metastatic LNs in regional LNs to accomplish complete elimination of residual metastatic LNs with leaving nonmetastatic LNs. In clinical settings, it should be useful for surgeons to employ PET/CT to find metastatic LNs before surgery, and intraoperative rapid diagnosis for regional LNs also helps surgeons for the proper discrimination. Furthermore, optical image-guided cancer surgery is a promising technique to adequately determine tumor margins by tumor-specific targeting, potentially leading to complete resection of tumor tissues (48–50). Combining these techniques can achieve accurate discrimination of metastatic LNs and avoid excessive LN dissection.

A recent study of breast cancer patients reported that shared TCRs between draining LNs and primary tumors were often observed (51), probably due to the assay of metastatic LNs. We analyzed LNs without metastasis, and very few shared TCRs between LNs and primary tumors were observed in MSS/pMMR CRCs. Whereas one concern is that the LNs tested may not be tumor-associated LNs, we collected LN samples of CRCs based on the anatomical location of lymphatic flow. Another important aspect of tumor-associated LNs should be the reflection of the TME: increased frequencies of dysfunctional effector T cells and immune suppressive cells. PD-1^+^CD8^+^ T cells and terminally differentiated CD8^+^ T cells in proximal LNs tended to be higher compared with tonsils. Additionally, Tregs were also more abundant in proximal LNs than in tonsils. The frequencies of these dysfunctional effector T cells and immune suppressive cells were gradually decreased in distal LNs, supporting that the LNs in our study were draining LNs of CRCs. Similar results showing few shared TCRs in LNs without metastasis were also observed in another study of CRC (52). However, the mechanisms of considerable shared TCRs between LNLs and TILs, especially in MSI-H/dMMR CRCs, remains unclear. One plausible explanation is that MSI-H/dMMR CRCs generally harbor high TMB that are recognized by the immune system as neo-antigens, and T cells specific for these neo-antigens are elicited in regional LNs (53). Indeed, MSI-H/dMMR CRCs had high TMB, potential neo-antigens, in our present study. Another possibility is that micrometastases are present in these regional LNs, and shared T cell clones are highly present, as in breast cancers. If there were pathological metastases including micrometastases in LNs, such LNs were excluded from our analyses. While we are not completely able to exclude micrometastases, we carefully avoided to include metastatic LNs in our nonmetastasis regional LNs as much as possible. The detailed mechanisms should be elucidated by further basic and translational studies.

In conclusion, we systemically analyzed the immunological status of tumors, nonmetastatic LNs, and peripheral blood in CRC patients, showing several differences in immunological status in each site and very few shared TCRs between LNs and primary tumors, especially those from MSS/pMMR CRC patients. By contrast, substantial shared TCRs were detected between nonmetastatic LNs and primary tumors in MSI-H/dMMR CRCs with high TMB. Furthermore, in our MSI-H/dMMR cohort, intensive LN dissection could provide negative impacts on long-term prognosis, suggesting that regional LNs play an important role in developing antitumor immunity against MSI-H/dMMR CRCs and that careful consideration needs to be paid regarding nonmetastatic LN dissection, particularly in MSI-H/dMMR CRC patients. However, since this was a hypothesis-generating study with a small patient cohort for whom we had an appropriate data set with a long follow-up, there are several limitations, including confounding factors in the clinical data, indicating the importance of future clinical studies with well-controlled large patient cohorts. Furthermore, technologies such as single-cell RNA sequencing and cellular index of transcriptomes and epitopes by sequencing (CITE-seq) may provide further findings on the shared T cell clones in MSI-H/dMMR CRCs, leading to optimal therapies against MSI-H/dMMR CRCs (54, 55).

## Methods

### Patients and samples.

Immunological phenotypes of paired PBLs, LNLs, and TILs from 21 CRC patients who underwent surgical resection at NCCHE between 2017 and 2020 were examined (NCCHE cohort). All surgeries were performed according to the JSCCR guidelines (17). Disease staging was performed by experienced pathologists using resected samples, including all dissected LNs according to the TNM classification (Union for International Cancer Control; UICC). Each dissected LN was halved with the maximum surface, all of which were pathologically examined, and all (not chosen by pathologists) remaining halved nonmetastatic LNs with sufficient cell numbers were subjected to immunological assays to avoid sampling bias. If there were pathological micrometastases in LNs, such LNs were excluded from the analyses. Well-differentiated, moderately differentiated, and poorly or undifferentiated tumors were defined according to the WHO guidelines. Tumors were classified as right colon if they were found in the cecum, ascending colon, or transverse colon and as left colon if they were in the descending colon, sigmoid colon, or rectum. LNs around marginal artery close to the primary tumors were defined as proximal LNs. When primary tumors were located in the right side, LNs around main trunk of the superior mesenteric artery were defined as distal LNs. When primary tumors were located in the left side, LNs around main trunk of the inferior mesenteric artery were defined as distal LNs. Both nonmetastatic proximal and distal LNs with sufficient cell numbers were subjected to the assays. PBLs were isolated by density gradient centrifugation with Ficoll-Paque (GE Healthcare). To collect LNLs and TILs, LNs and tumor tissues were minced and treated with gentleMACS Dissociator (Miltenyi Biotec) (56). In addition, tonsils were provided from patients with chronic tonsillitis who underwent surgical resection at Nagoya University Hospital as control.

In addition, patients with pStage 0–III MSI-H CRC who received surgical resection at SCC between 1999 and 2015 were enrolled in the prognostic analysis as the MSI-H cohort (SCC cohort). All patients who were eligible for surgical resection had a good performance status. These analyses were retrospectively performed.

### IHC for MMR status.

MMR status was examined with IHC with an anti–mutL homolog 1 (MLH1; ES05) mAb, anti–mutS homolog 2 (MSH2; FE11) mAb, anti–postmeiotic segregation increased 2 (PMS2; EP51) mAb, and anti–mutS homolog 6 (MSH6; EP49) mAb (Dako). Tumors were considered negative for MLH1, MSH2, PMS2, or MSH6 expression only if there was a complete absence of nuclear staining in tumor cells, and normal epithelial cells and lymphocytes were used as internal controls. Tumors lacking MLH1, MSH2, PMS2, or MSH6 expression were defined as dMMR, whereas tumors that maintained expression of all markers were considered pMMR.

### Microsatellite instability status.

MSI analysis was performed using fluorescence-based PCR as described previously (57). Briefly, MSI status was determined using 5 Bethesda markers (BAT25, BAT26, D5S346, D2S123, and D17S250) and classified as MSI-H (when 2 or more markers were demonstrated to be unstable), MSI-low (MSI-L; when only 1 marker was unstable), and MSS (when no markers were unstable). MSI-positive markers were reexamined at least twice to confirm the results.

### WES.

DNA was extracted from available frozen tumor samples and paired blood samples using a QIAmp DNA Mini Kit (QIAGEN) according to the manufacturer’s instructions. Sequencing libraries were prepared for WES with an NEBNext Ultra DNA Library Prep Kit (New England BioLabs) according to the manufacturer’s instructions. Adaptor-ligated samples were amplified with 6 PCR cycles. Amplified DNA fragments underwent enrichment of exonic fragments using a SureSelect Human All Exon Kit v5 (Agilent Technologies). Massively parallel sequencing of isolated fragments was performed with a Novaseq6000 (Illumina). Paired-end WES reads were independently aligned to the human reference genome (hs37d5) using BWA-MEM (58). Picard MarkdDuplicates (http://broadinstitute.github.io/picard/) was used to remove PCR duplicates. The resultant BAM files were processed using GATK tools (59). Local realignments of insertions and deletion was performed using GATK IndelRealigner. Systematic errors in base quality scores were corrected using GATK BaseRecalibrator. Somatic mutations and short indels were called using GATK MuTect2 (http://www.broadinstitute.org/cancer/cga/mutect). Variants calls from Mutect2 filtered with GATK FilterMutectCalls. Mutations were discarded manually if the mutant allele read depth was < 5, the variant allele frequency was < 0.1, or the variant base was observed in the normal tissue. Gene mutations were annotated by ANNOVAR (60). The data were deposited in the Japanese Genotype-phenotype Archive (accession no. JGAD000385; https://ddbj.nig.ac.jp/resource/jga-dataset/JGAD000385).

### Immunological phenotype analyses.

Flow cytometry assays were performed as described (56). The antibodies used in the flow cytometry analyses are summarized in Supplemental Table 3. Briefly, cells were washed with PBS containing 2% FCS and subjected to staining with surface antibodies. Intracellular staining of FOXP3 was performed with an anti-FOXP3 mAb and the FOXP3/Transcription Factor Staining Buffer Set (Thermo Fisher Scientific) according to the manufacturer’s instruction. After washing, cells were analyzed with an LSR Fortessa instrument (BD Biosciences) and FlowJo software (BD Biosciences). Dilution of the staining antibodies followed the manufacturer’s instructions.

### 16S rRNA sequencing.

A DNeasy PowerSoil Kit (QIAGEN) was used to extract DNA from fecal samples. Bacterial community analysis targeting the V1-V2 region (27Fmod, 5′ - AGR GTT TGA TCM TGG CTC AG -3′; 338R, 5′- TGC TGC CTC CCG TAG GAG T - 3′) of the 16S rRNA gene was performed on a MiSeq platform (Illumina). The bioinformatics pipeline QIIME, version 1.9.1, was utilized as the informatics environment for all relevant processing of raw sequencing data and the calculation of bacterial relative abundances.

### TCR sequencing.

TCR usage was analyzed as previously reported (61). Briefly, total RNA was prepared from paired proximal LNs and primary tumors. Complementary DNA was synthesized from total RNA, and TCR β chains were amplified using adaptor ligation–mediated PCR. Then, using the PCR products as templates, TCR sequences were analyzed using Miseq according to the manufacturer’s protocol. Alignments among approximately 10,000 sequences/run were performed with IMGT/V-QUEST (http://www.imgt.org).

### Public data analyses.

Differentially expressed genes between MSS and MSI-H CRCs were extracted from TCGA data sets using fold changes with FDR by edgeR, and CIBERSORTx was performed as previously reported (12, 18, 62).

### Statistics.

The relations of continuous variables between or among groups were compared with *t* test or 1-way ANOVA, respectively. For multiple testing, Bonferroni correction was employed. The univariate relationship between each independent variable was examined using the Fisher’s exact test. RFS was defined as the time from surgery until the first observation of disease progression. A ROC curve of LN dissection number for recurrence within 2 years after surgery was constructed to determine a cut-off value. The RFS was investigated with the Kaplan-Meier method and was compared among groups using the log-rank test. All tests were 2 tailed, and *P* values less than 0.05 were considered statistically significant. Statistical analyses were performed using Prism version 7 software (GraphPad Software Inc.).

### Study approval.

This study was approved by the IRB of the National Cancer Center (nos. 2015-048 and 2016-029), Nagoya University (no. 2016-0159), and SCC (no. 465) and was conducted in accordance with international ethics guidelines, including the Declaration of Helsinki. All donors provided written informed consent before sampling in accordance with the Declaration of Helsinki. This study was performed in a blinded and nonrandomized manner. All surgeries were performed according to the JSCCR guidelines (17). We obtained written informed consent from all participants before sampling for immunological analyses.

## Author contributions

Study concept and design were contributed by YT and HN. Development of methodology was contributed by KI, YT, KO, DM, and HN. Acquisition of data was contributed by KI, YT, SF, KO, DM, YK, DS, and MK. Analysis and interpretation of data were contributed by KI, YT, SF, KA, KO, TI, DM, YK, DS, MK, NS, SN, YS, SM, MI, and HN. Writing, review, and/or revision of the manuscript were contributed by KI, YT, SF, KA, KO, TI, DM, YK, DS, MK, NS, SN, YS, SM, MI, and HN. Study supervision was contributed by YT, NS, MI, and HN. All authors read and approved the final manuscript.

## Supplementary Material

Supplemental data

## Version 1 03/23/2021

In-Press Preview

## Version 2 05/10/2021

Electronic publication
