# Supplementary material for: Importance of lymph node immune responses in MSI-H/dMMR colorectal cancer
Source: JCI Insight. 2021 May 10;6(9):e137365. doi: 10.1172/jci.insight.137365 (PMC8262295; doi:10.1172/jci.insight.137365)
Supplement: Supplemental data [file jciinsight-6-137365-s066.pdf]

Supplemental Figure 1. LN dissection numbers according to tumor location.

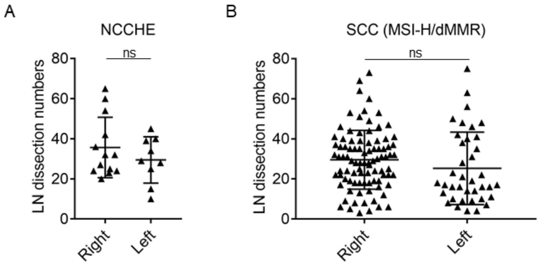

LN dissection numbers according to tumor location (right vs. left) are summarized in 21 NCCHE CRC cohort (**A**) and 130 SCC MSI-H/dMMR CRC cohort (**B**), respectively. Means and SDs are shown, and statistical analyses were performed using the t-tests. ns, not significant.

Supplemental Figure 2. Clustering with T cell phenotypes in TILs.

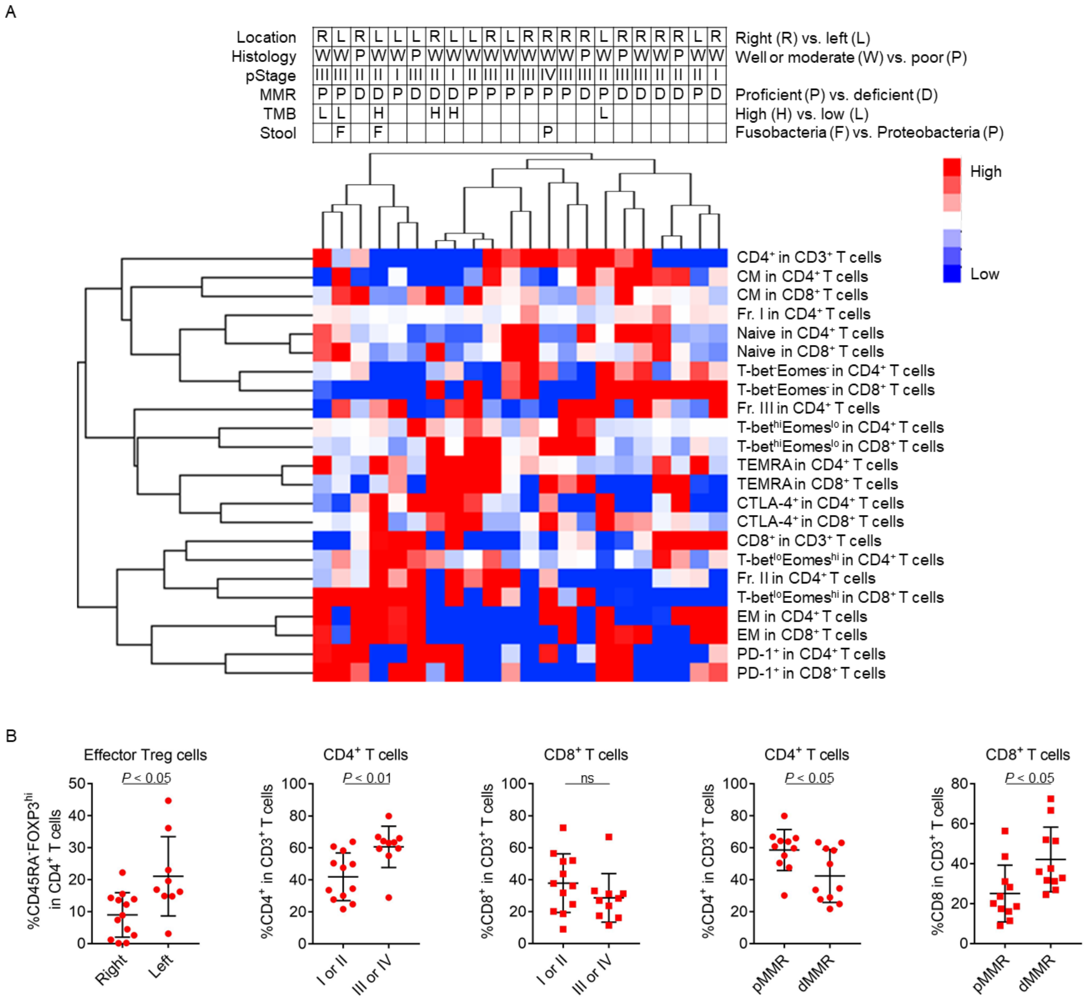

Twenty-two TILs from 21 CRC patients were examined with flow cytometry as in Figure 1. Clustering with T cell phenotypes in the TME with clinicopathological features (**A**), and the frequencies of eTreg cells in the TME according to tumor location, $CD4^+$ T cells and $CD8^+$ T cells in the TME according to pStage, and $CD4^+$ T cells and $CD8^+$ T cells in the TME according to MMR status (**B**) are shown. Means and SDs are shown, and statistical analyses were performed using the t-tests.

Supplemental Figure 3. Clustering with T cell phenotypes in LNLs.

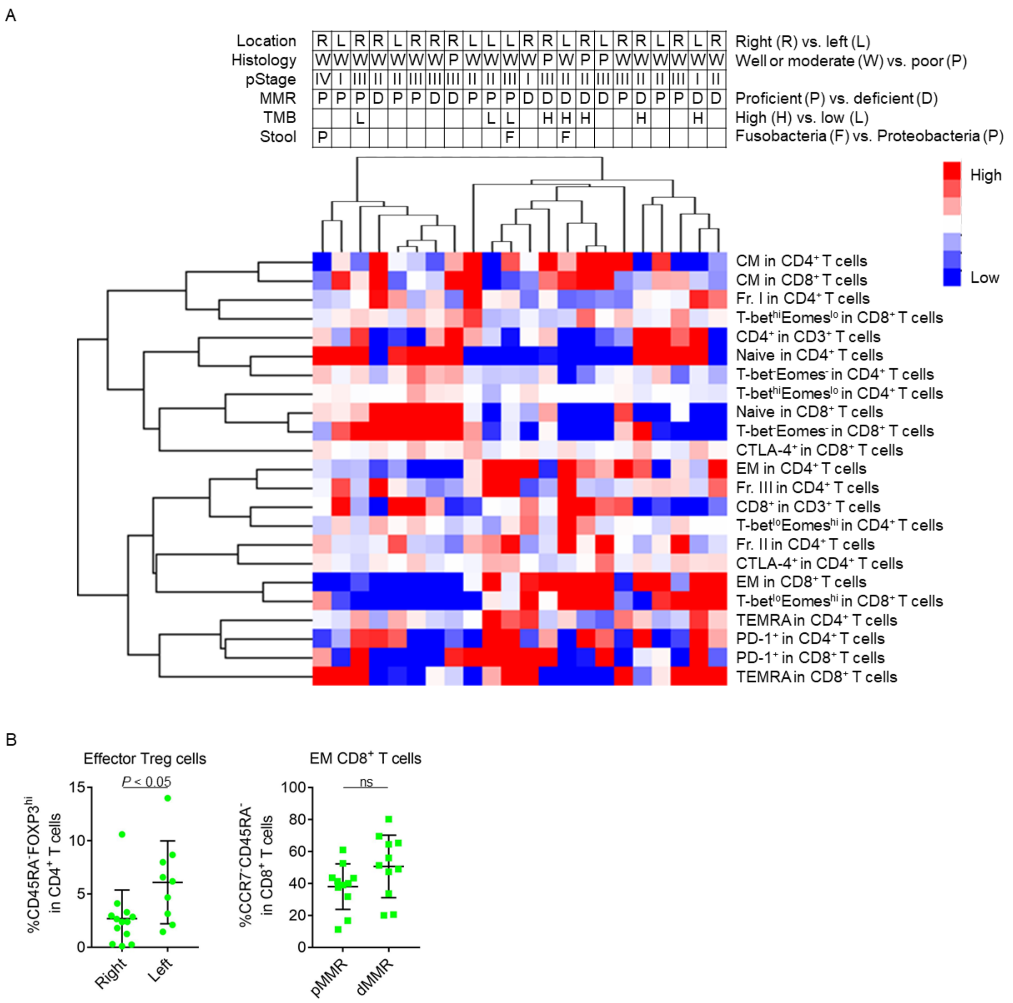

Twenty-two LNLs of proximal LNs from 21 CRC patients were examined with flow cytometry as in Figure 1. Clustering with T cell phenotypes in proximal LNs with clinicopathological features (**A**), and the frequencies of eTreg cells in LNLs according to tumor location and effector memory CD8^{+} T cells in LNLs according to MMR status (**B**) are shown. Means and SDs are shown, and statistical analyses were performed using the t-tests. EM, effector memory; ns, not significant.

Supplementary Figure 4. Analyses of TCGA datasets of CRCs.

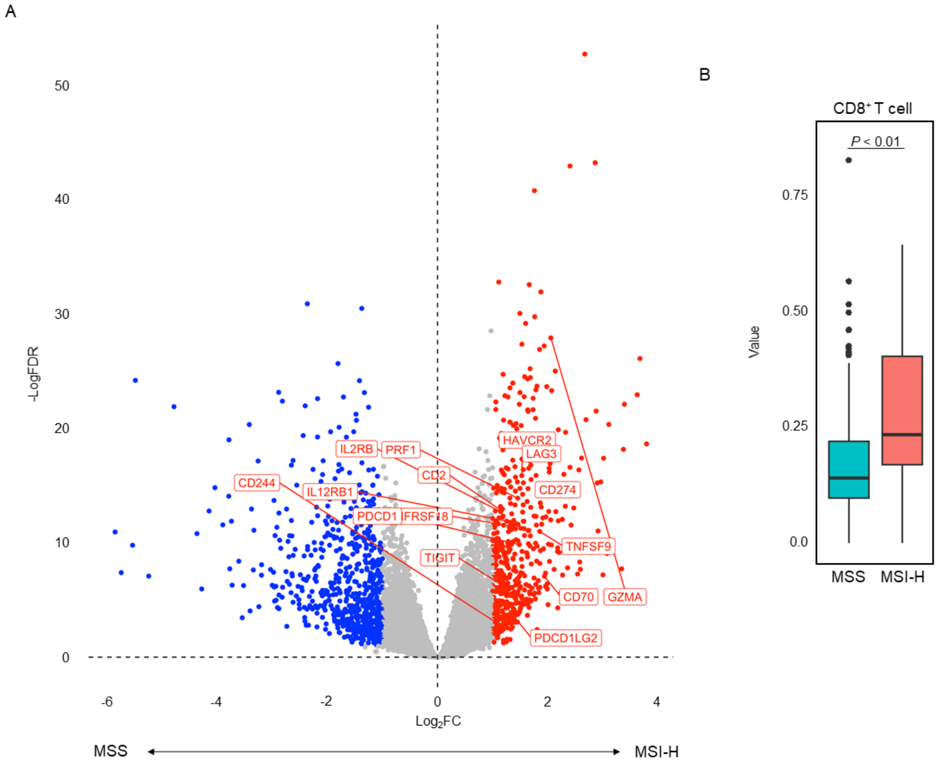

(A) Volcano plots according to microsatellite status. TCGA datasets of CRCs were examined, and differentially expressed genes between MSS and MSI-H were extracted. FC, fold change; FDR, false discovery rate. **(B)** CD8^{+} T cell infiltration according to microsatellite status. TCGA datasets were analyzed with CIBERSORTx. Statistical analysis was performed using the t-tests.

Supplemental Figure 5. The proportion of CD4$^+$ T cells and CD8$^+$ T cells in PBLs, LNLs and TILs.

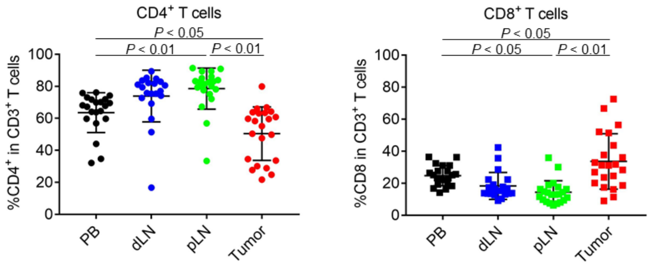

PBLs, LNLs and TILs from 21 CRC patients were examined with flow cytometry as in Figure 1. Summaries for the frequencies of CD4$^+$ T cells and CD8$^+$ T cells are shown. Means and SDs are shown, and statistical analyses were performed using the one-way ANOVA tests with Bonferroni corrections. PB, peripheral blood; dLN, distal LN; pLN, proximal LN; ns, not significant.

Supplemental Figure 6. Comparison of immunological phenotypes between tonsils and LNs.

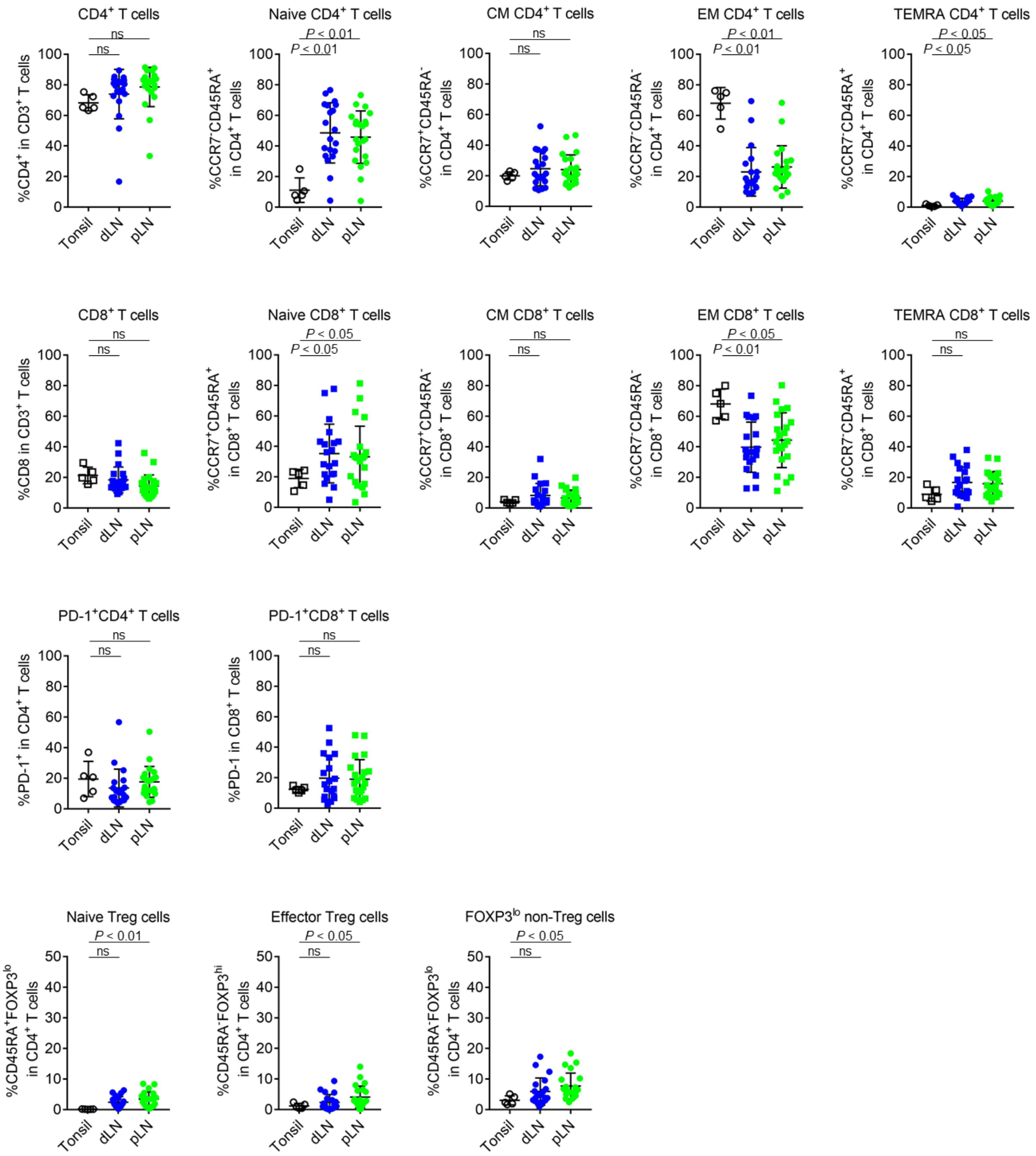

Tonsils from 5 patients with chronic tonsillitis who received surgical resection of tonsils were minced like tumor tissues, and immunological phenotypes were examined with flow cytometry. T cell fractions indicated were compared between tonsils and LNs. Means and SDs are shown, and statistical analyses were performed using the one-way ANOVA tests with Bonferroni corrections. dLN, distal LN; pLN, proximal LN; ns, not significant.

Supplemental Figure 7. T-bet and Eomes expression in $CD4^+$ T cells.

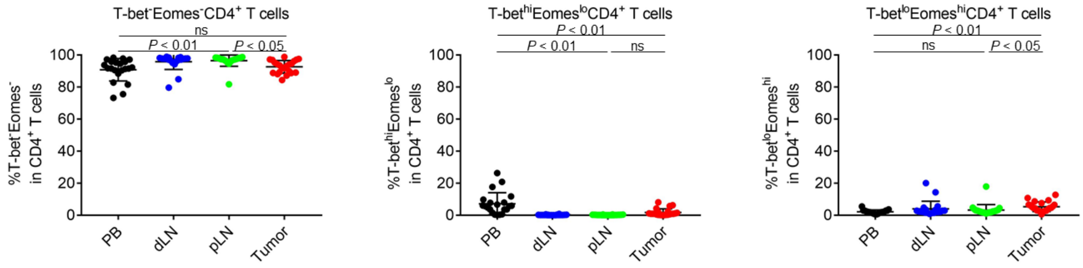

PBLs, LNLs and TILs from 21 CRC patients were examined with flow cytometry as in Figure 1. Summaries for frequencies of T-bet and Eomes expressing cells in $CD4^+$ T cells in PBLs, LNLs and TILs are shown. Means and SDs are shown, and statistical analyses were performed using the one-way ANOVA tests with Bonferroni corrections. PB, peripheral blood; dLN, distal LN; pLN, proximal LN; ns, not significant.

Supplemental Figure 8. The expression of Treg cell-related molecules by each FOXP3$^+$CD4$^+$ T cell subpopulation.

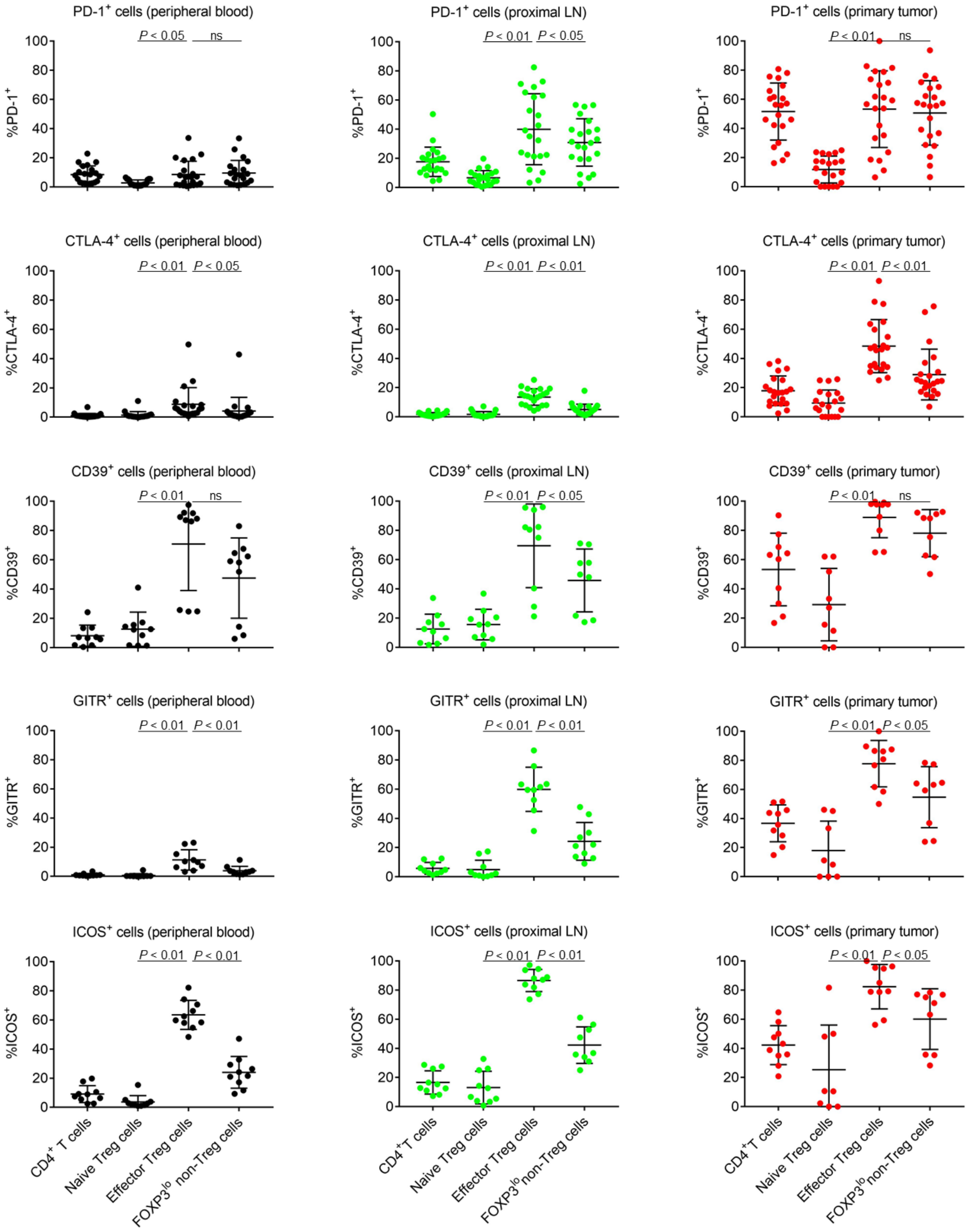

PBLs and LNLs of proximal LNs from CRC patients were examined with flow cytometry as in Figure 1. Summaries for frequencies of PD-1, CTLA-4, CD39, GITR and ICOS expressing cells in total CD4$^+$ T cells, naive Treg cells, eTreg cells and FOXP3^{low} non-Treg cells in PBLs, LNLs and TILs are presented. Means and SDs are shown, and statistical analyses were performed using the one-way ANOVA tests with Bonferroni

corrections. ns, not significant.

Supplemental Figure 9. The proportion of FOXP3$^-$CD4$^+$ T cell subpopulation.

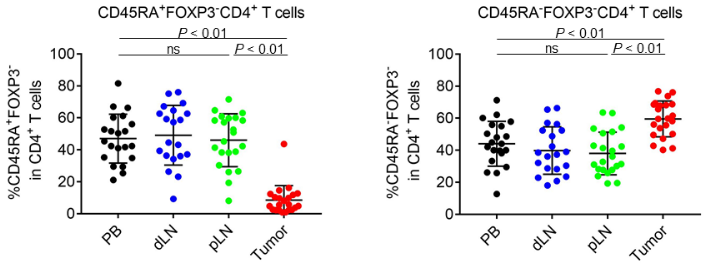

PBLs, LNLs and TILs from 21 CRC patients were examined with flow cytometry as in Figure 1. Summaries for frequencies of CD45RA$^-$FOXP3$^-$CD4$^+$ T cells and CD45RA$^+$FOXP3$^-$CD4$^+$ T cells in CD4$^+$ T cells are shown. Means and SDs are shown, and statistical analyses were performed using the one-way ANOVA tests with Bonferroni corrections. PB, peripheral blood; dLN, distal LN; pLN, proximal LN; ns, not significant.

Supplemental Figure 10. TCR analyses according to MMR status and pathological staging.

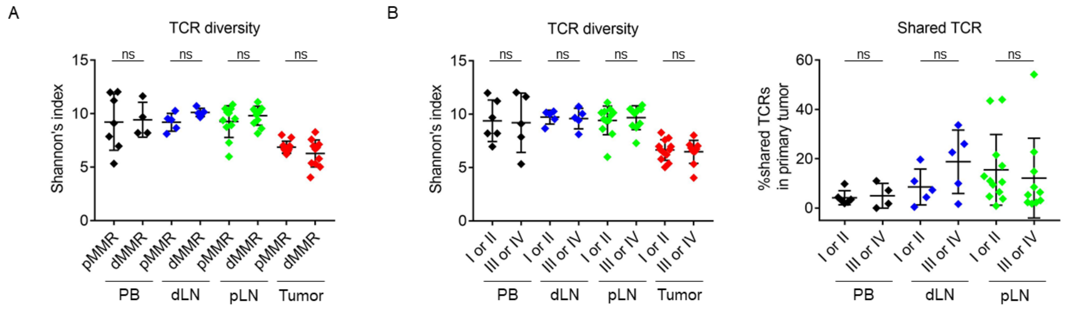

PBLs, LNLs and TILs from 21 CRC patients who received surgical resection were prepared, and the TCR sequencing was performed with next-generation sequencing. If there were remaining samples, the TCR sequencing was performed for PBLs and LNLs of distal LNs. **(A)** Diversity of TCR repertoire according to MMR status. The diversity was evaluated with Shannon's index. **(B)** Diversity of TCR repertoire and shared TCRs in TILs according to pathological staging. Shannon's index (left) and the frequency of shared TCRs in TILs (right) of each sample are shown. Means and SDs are shown, and statistical analyses were performed using the t-tests. PB, peripheral blood; dLN, distal LN; pLN, proximal LN; ns, not significant.

Supplemental Figure 11. TMB of pMMR and dMMR CRCs.

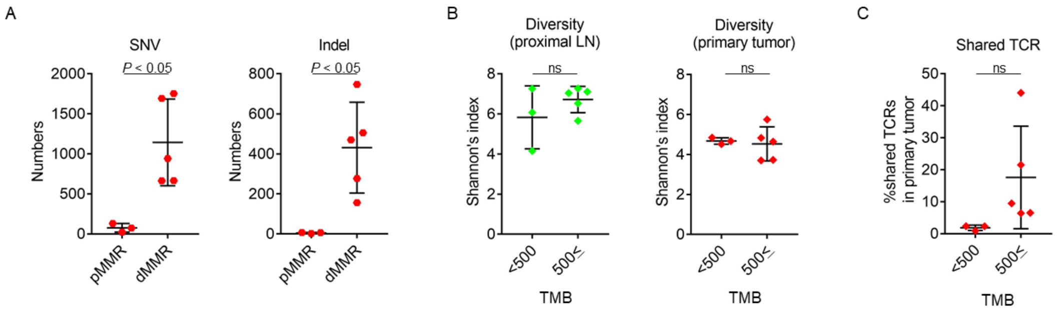

TMB was analyzed with WES using next-generation sequencing if remaining tumor DNA samples were available. Comparison in non-synonymous single nucleotide variations (SNV) (left) and insertion or deletion (indel) (right) between pMMR and dMMR CRCs (**A**), the associations between TMB and TCR diversity in proximal LN (left) and tumors (right) (**B**) or between TMB and shared TCRs in LNLs and TILs (**C**) are presented. Means and SDs are shown, and statistical analyses were performed using the t-tests. ns, not significant.

Supplemental Figure 12. An ROC curve of LN dissection number for recurrence.

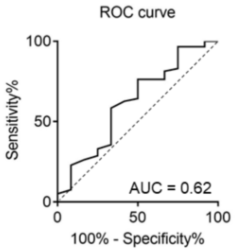

An ROC curve of LN dissection number for recurrence within 2 years after surgery was constructed to determine the cut-off value. ROC, receiver operating characteristic; AUC, area under curve.

Supplemental Table 1. Patient characteristics subjected to immunological analyses (NCCHE cohort).

Characteristics (n = 21, 22 samples)	pMMR (n = 11, 11 samples)	dMMR (n = 10, 11 samples)	*P*
Age (years)			
Average ± SD (median)	60.2 ± 11.9 (61.5)	65.6 ± 13.9 (67)	0.022
Sex			
Male	5	7	0.39
Female	6	3	
Tumor location			
Right	5	8	0.39
Left	6	3	
Differentiation			
Well or moderate	11	6	0.035
Poor	0	4	
pStage			
I	1	2	
II	4	5	0.67¶
III	5	4	
IV	1	0	
LN dissection number			
Average ± SD (median)	34.6 ± 14.4 (30.5)	32.9 ± 14.7 (28)	0.73

¶pStage I or II vs. III or IV

Supplemental Table 2. Patient characteristics of the MSI-H cohort (SCC cohort).

**Characteristics**	**n = 130**
Age (years)	
Average ± SD (median)	64.8 ± 12.5 (66)
Sex	
Male	56
Female	74
Tumor location	
Right	39
Left	91
Differentiation	
Well or moderate	31
Poor	99
pStage	
0	2
I	29
II	68
III	31
Tumor diameter	
Average ± SD (median)	57.2 ± 34.9 (50)
Metastatic LN number	
Average ± SD (median)	0.76 ± 1.88 (0)
LN dissection number	
Average ± SD (median)	28.3 ± 15.8 (28)

Supplemental Table 3. Summary of antibodies used in flow cytometry analyses.

Tag	Molecule	Clone	Company
AF700	CD3	UCHT1	Thermo Fisher Science
V500	CD4	RPA-T4	BD Biosciences
BV785	CD8a	RPA-T8	BioLegend
BV605	CCR7	G043H7	Biolegend
BV711	CD45RA	HI100	BD Biosciences
PE-Cy7	T-bet	4B10	Thermo Fisher Science
FITC	Eomes	WD1928	Thermo Fisher Science
PE	FOXP3	236A/E7	Thermo Fisher Science
BV421	PD-1	MIH4	BD Biosciences
APC	CTLA-4	L3D10	Biolegend
BV605	CD39	TU66	BD Biosciences
PE-Cy7	GITR	eVioAITR	Thermo Fisher Science
APC	ICOS	ISA-3	Thermo Fisher Science
